# Gastrointestinal Tolerance and Glycemic Response of Isomaltooligosaccharides in Healthy Adults

**DOI:** 10.3390/nu10030301

**Published:** 2018-03-03

**Authors:** Vishnupriya Gourineni, Maria L. Stewart, Didem Icoz, J. Paul Zimmer

**Affiliations:** 1 Global Nutrition R&D, Ingredion Incorporated, 10 Finderne Ave, Bridgewater, NJ 08807, USA; maria.stewart@ingredion.com (M.L.S.); paul.zimmer@bayer.com (J.P.Z.); 2 Global Sweeteners R&D, Ingredion Incorporated, 10 Finderne Ave, Bridgewater, NJ 08807, USA; didem.icoz@ingredion.com

**Keywords:** isomaltooligosaccharides, glycemic response, breath hydrogen, tolerance

## Abstract

Ingredients delivering functional and nutritional benefits are of interest to food manufacturers. Isomaltooligosaccharides (IMOs) which serve as alternate sweeteners fit into this category. IMOs are a mixture of α-(1 → 6) and α-(1 → 4)-linked glucose oligomers, synthesized by an enzymatic reaction from starch (corn, tapioca). The aim of this study was to evaluate the fermentability and glycemic response of IMO in a healthy population. Two randomized, double-blind, placebo-controlled, cross-over human studies were conducted. In the first study (*n* = 26), participants’ breath hydrogen over 24 h, gastrointestinal tolerance, and glycemic and insulinemic response to BIOLIGO^TM^ IL5040 isomaltooligosaccharide were measured. In another study (*n* = 10), participants’ two-hour post-prandial glycemic response to BIOLIGO^TM^ IL5040 isomaltooligosaccharide and BIOLIGO^TM^ IL7010 isomaltooligosaccharide was measured compared to dextrose (control). The IMOs differed in the composition of mono and di-saccharide sugars. IMO syrup dose was matched for 50 g of total carbohydrates and was consumed by mixing in water (237 mL/8 oz.). Mean composite gastrointestinal score was not significantly different (*p* = 0.322) between the control (1.42) and IMO (1.38). Lack of difference in glycemic response (*p* = 0.662), with no impact on breath hydrogen (24 h; *p* = 0.319) and intestinal tolerance, demonstrates that IMO is digestible and can be used to replace sugars in product formulations.

## 1. Introduction

Global forecasts on obesity and diabetes prevalence are among the top issues of concern, and they have led to increased scrutiny of sugar-sweetened products [1]. The World Health Organization (WHO) recommends limiting sugar intake to 10% of total energy intake, equivalent to 12 teaspoons or 50 g/day [2]. This has been adopted by various nations, with public policies restricting added sugars through taxes on sugar beverages and warning labels. More recently, the US Food and Drug Administration (FDA) mandated the inclusion of “added sugars” on the nutrition facts labeling. Although there is no consistent evidence that added sugars cause weight gain leading to obesity in children and adults, consumer surveys point to the heightened market need for sugar alternatives in full-calorie products [3,4,5].

One such alternative is sugar alcohols (polyols). Polyols provide sweet taste while yielding a low glycemic index, are non-cariogenic, and thus can be used as sugar replacers [6]. However, polyols use at higher levels is limited due to their effects on gastrointestinal intolerance in healthy subjects and patients with irritable bowel syndrome (IBS) [7,8]. Maltitol, at higher levels, caused intestinal discomfort by inducing osmatic pressure, but when combined with short-chain fructooligosaccharide, a prebiotic fiber, has been shown to attenuate the intestinal symptoms in healthy adults [9]. More commonly used alternate sweeteners in Asia are fructooligosaccharides (FOS) and isomaltooligosaccharides (IMO). Oligosaccharides are appealing as alternate sweeteners due to their improved functionality and nutritional benefits [10]. Fructooligosaccharides are unique in a way providing both nutritional (fiber) and functional (sweetness) benefits when formulated in food and beverages. However, the use of FOS has few limitations with regard to its effect on digestive intolerance at higher doses and lack of stability in highly acidic products. The physicochemical properties of isomaltooligosaccharides make them highly functional as sweeteners, due to their higher stability in food and beverage formulations, compared to FOS [11].

Isomaltooligosaccharides are mixtures of α-(1 → 6)-linked glucose oligomers with degrees of polymerization from 2–10, and their carbohydrate composition includes isomaltose, panose, isomaltotriose, isomaltotetraose, isomaltopentaose and so forth. IMOs are functional sweeteners and commonly derived from the enzymatic processing of starch (transglycosylation of hydrolyzed starch from corn and tapioca being the most commonly used bases) on a commercial scale. Its sweetness depends on the composition, specifically on the amount of lower molecular weight components, such as glucose (about 70–75% as sweet as sucrose) and maltose (about 30–35% as sweet as sucrose). IMOs can also be produced via bacterial fermentation of sucrose in the presence of a maltose acceptor by a glucosyltransferase enzyme, such as dextransucrase [12]. In addition to α-(1 → 6)-linked glucose oligomers, commercial IMO products also contain some level of α-(1 → 4)-linked glucose oligomers, including maltose, maltotriose, etc. [13]. Rarely, it is possible to find minor amounts of α-(1 → 2) and α-(1 → 3)-linked kojibiose and nigerose in the products as well. IMOs naturally exist in honey, and fermented foods, such as soy sauce, miso, and sake.

Although IMOs are promoted as prebiotic fiber in Asia, there is conflicting evidence on their digestibility with high caloric value as shown in rat and human studies [14,15]. Madsen et al. [12] have surveyed a number of commercially available IMO products in the US, and their results indicated that the digestibility and potential glycemic impact of these ingredients were inconsistent with product labels, including soluble fiber content and glycemic response. Our aim was to assess gastrointestinal tolerance and glycemic response of two IMOs, BIOLIGO^TM^ IL5040 and BIOLIGO^TM^ IL7010, in healthy individuals. In the first study, breath hydrogen response, gastrointestinal tolerance, glycemic and insulinemic response to BIOLIGO^TM^ IL5040 IMO was tested. In the second study, two-hour glycemic response to BIOLIGO^TM^ IL5040 and BIOLIGO^TM^ IL7010 IMOs was evaluated.

## 2. Materials and Methods 

Two IMO syrups manufactured by Ingredion Incorporated (Bridgewater, NJ, USA) were used in these studies. The first product BIOLIGO™ IL5040 contains 50% IMO, and 40% mono- and disaccharides, consisting of mainly dextrose and maltose, and a small amount of isomaltose. The second product BIOLIGO™ IL7010 is a reduced mono- and disaccharide version of BIOLIGO™ IL5040, and contains 70% IMO, and less than 10% mono- and disaccharides. IMO content for these products is defined as the IMO components from DP2 to DP7 (isomaltose, panose, isomaltotriose, isomaltotetraose, isomaltopentaose, isomaltohexaose, isomaltoheptaose).

The studies were conducted in accordance with the ethical principles outlined in the Declaration of Helsinki (2000), Good Clinical Practice Guidelines, and the United States 21 Code of Federal Regulations. Study 1 was conducted at MB Clinical Research Labs (Glen Ellyn, IL, USA) in 2016, and protocol approved by the Hummingbird institutional review board (Cambridge, MA, USA; IRB number # MB-1518). Study 2 was conducted by The Glycemic Index Laboratories (Toronto, ON, Canada) in 2014, and protocol approved by the Western Institutional Review Board (Vancouver, BC, Canada; IRB number # 441 WIRB). All subjects provided written informed consent prior to starting the study.

### 2.1. Subject Screening

#### 2.1.1. Study 1

*Inclusion criteria*: Healthy men or women aged 18 to 75 years, body mass index (BMI) 18.50–29.99 kg/m^2^, normally active and judged to be in good health on the basis of their medical histories, were enrolled in the study. 

*Exclusion criteria:* Subjects were excluded if they had fasting capillary glucose ≥100 mg/dL at screening; major trauma or a surgical event within 3 months of screening; history of drug or alcohol abuse; have body weight change ≥4.5 kg in the 2 months prior to screening; uncontrolled hypertension; use of antibiotics; symptoms of an active infection; intolerance to any ingredients in the study products; extreme dietary habits; cannot abstain from consuming probiotics; alcohol; smoking, and who are unwilling to comply with the experimental procedures.

#### 2.1.2. Study 2

*Inclusion criteria*: Subjects were males or non-pregnant females aged 18–75 years and in good health.

*Exclusion criteria:* Subjects less than 18 years old or older than 75 years, with a known history of AIDS, hepatitis, diabetes or a heart condition, and unwillingness or inability to comply with the experimental procedures, and to follow GI Labs safety guidelines.

### 2.2. Study Design and Test Products

Study 1: The study was a randomized, double-blinded, placebo-controlled, cross-over design, with 26 healthy adults (age 18–75 years, body mass index (BMI) 18.50–29.99 kg/m^2^). Eligible participants were tested on separate days and were assigned randomly to either BIOLIGO^TM^ IL5040 IMO (test, 68.46 g), or dextrose (CERELOSE^®^ Dextrose, Ingredion Incorporated, Bridgewater, NJ, USA) (control; 54.77 g) mixed in 237 mL (8 oz) of water. Both the test and control were matched for 50 g total carbohydrates. The interval between two testing periods was one week. Of 31 randomized subjects, five did not meet inclusion/exclusion criteria and 26 participants completed the study.

Study 2: In a double-blind, randomized crossover design, eligible participants (*n* = 10) were studied on three separate days, over a period of 2 to 3 weeks with an interval of no less than one day between tests. A total of 10 subjects were randomly assigned to either BIOLIGO^TM^ IL5040 IMO (66.3 g); BIOLIGO^TM^ IL7010 IMO (66.0 g), or dextrose (Clintose^®^ Dextrose, ADM, Chicago, IL, USA) (control; 54.9 g) mixed in 250 mL of water. The test ingredients and control were matched for 50 g of total carbohydrates.

The doses of the IMOs were based on the batch certificate of analysis, and slight differences in BIOLIGO^TM^ IL5040 IMO doses between the two studies are due to differences in water content.

### 2.3. Study Visit Procedures

Study 1: On the test day, all participants arrived at the clinic after an overnight fasting. The initial breath hydrogen, fasting blood glucose and insulin were measured and participants were provided either IMO or dextrose (control) mixed in water. Blood samples were obtained for serum glucose and insulin measurements via an indwelling venous catheter or venipuncture at *t* = −15, 15, 30, 45, 60, 90, 120, 150, 180, 210, and 240, where *t* = 0 min was the start of study product consumption. Carbohydrate malabsorption and fermentation were measured by assessing each subject’s end-alveolar (breath) hydrogen concentrations on test days, following the *t* = −15 (pre-dose), 60, 120, 180, and 240 min blood collections. Additionally, a breath sample was collected in the clinic at *t* = 24 h, and subjects collected breath samples at home at *t* = 8 and 12 h. During both test days, a low-fiber, very low-dairy standardized lunch was administered in the clinic on the study days, and a low-fiber, very low-dairy dinner, and evening snack were dispensed to be consumed at home that evening. 

A gastrointestinal tolerability (GI) questionnaire [16] was administered immediately after the test visits, to assess the presence and severity of selected GI symptoms including nausea, GI rumblings, abdominal pain, bloating, flatulence, and diarrhea over the past 24-h period. GI symptoms were scored as follows: 0 = none, 1 = no more than usual, 2 = somewhat more than usual, and 3 = much more than usual. A composite score was also calculated as the sum of the six individual GI symptom ratings, for a total possible score of 0–12.

Study 2: On each test occasion, after subjects were weighed, two fasting blood samples were obtained by finger-prick at 5-min intervals. Following consumption of either IMO or dextrose (control) in water, blood samples were collected at 15, 30, 45, 60, 90, and 120 min.

### 2.4. Biochemical Analysis

Study 1: Glucose was measured using an enzymatic colorimetric method—GOD/PAP Method (Randox Laboratories Ltd., Kearneysville, WV, USA) utilizing glucose oxidase and peroxidase to degrade into Phenol, and 4-Aminoantipyrine, measured using Trinder indicator reaction at 505 nm. The increase in absorbance correlates with the glucose concentration of the sample with an analytical coefficient of variation (CV) of <2%. Insulin was measured with an immunoturbidimetry assay (Kamiya Biomedical Company, Seattle, WA, USA). A radioimmunoassay method for measuring insulin (HI-14K, Millipore Corporation, Billerica, MA, USA), was conducted at the University of California, Davis (Davis, CA, USA) in a subset of hemolyzed, and non-hemolyzed samples. A regression equation was developed to convert radioimmunoassay values to immunoturbidimetric values. Breath samples of end-alveolar air were collected into 10 mL glass vacuum tubes using an EasySampler device (Quintron Instruments, Milwaukee, WI, USA). The concentrations of hydrogen in breath samples were analyzed by gas chromatography with a resolution of 1 ppm, and accuracy of ±2–3 ppm and a linear range: 2–150 ppm for hydrogen (Microlyzer Gas Analyzer, model SC; Quintron Instruments, Milwaukee, WI, USA).

Study 2: Blood glucose analysis was done using a YSI (Yellow Spring Instruments, Yellow Springs, OH, USA) analyzer, and took place within five days of collection. The YSI uses a wet method for glucose analysis based on the reaction of glucose in the sample with immobilized glucose oxidase. The typical analytical CV for fasting glucose is <2%.

### 2.5. Data Analysis and Statistics (Sample Size, Data Analysis and Statistical Analysis)

Study 1: A power analysis indicated that sample size of 26 subjects would be required to detect a 0.58 standard deviation difference between treatments for continuous outcome variables with 80% power, alpha = 0.05, 2-sided. A total of 31 subjects were randomized to allow for subject attrition.

Study 2: Using the t-distribution, and assuming an average CV of within-individual variation of incremental area under the curve (iAUC) values of 25%, *n* = 10 subjects has 80% power to detect a 33% difference in incremental AUC with 2 tailed *p* < 0.05. Paired *t*-tests were conducted on blood glucose, insulin, and values at individual and incremental area-under-curveusing GraphPad Prism 7 (v7.03, GraphPad Software, Inc., La Jolla, CA, USA). *p* values ≤ 0.05 were deemed statistically significant, and data are presented as mean ± SEM.

## 3. Results

Subject demographics for both studies are shown in Table 1 and Table 2. All participants were healthy in both studies. 

**Figure 1:**
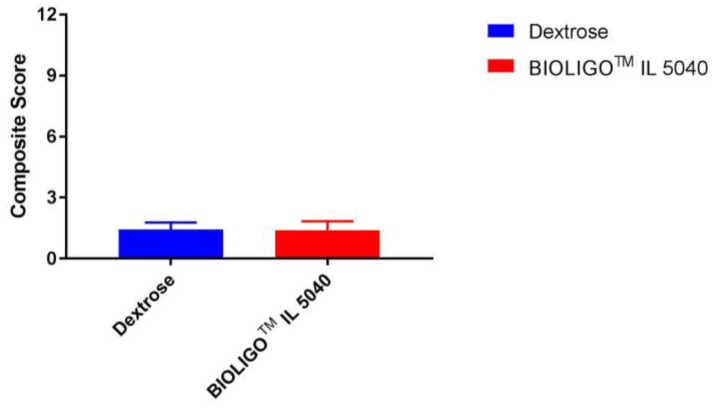
Composite gastrointestinal tolerance scores (Study 1).

Composite gastrointestinal tolerance in response to IMO and dextrose (control) is shown in Figure 1. There was no significant difference (*p* = 0.322) between the control (1.42) and IMO (1.38) in the mean composite score on the GI tolerability questionnaire. Similar findings were observed for individual gastrointestinal frequency of scores of 2 or greater (somewhat more than usual and much more than usual) on the components of the GI tolerability questionnaire as shown in Table 3.

GI symptoms (nausea, bloating, GI rumblings, flatulence, abdominal pain, diarrhea) were scored as follows: 0 = none, 1 = no more than usual, 2 = somewhat more than usual and 3 = much more than usual. The composite score was the sum of the six individual GI symptom ratings. Data are mean ± SEM.

**Figure 2:**
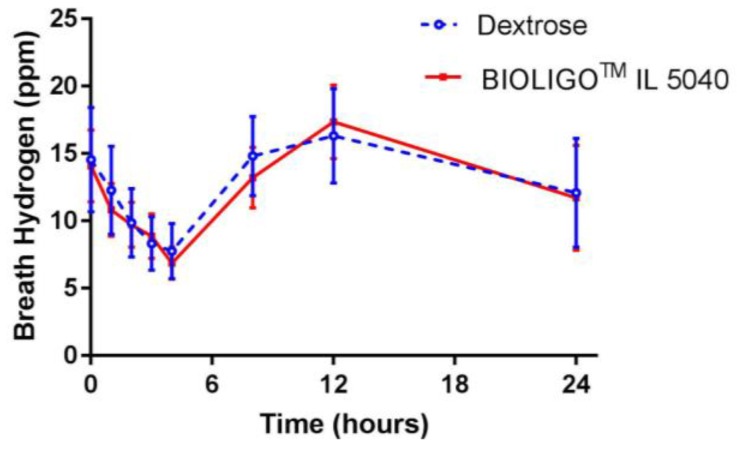
Breath hydrogen (ppm) measured over 24 h (Study 1); Data are mean ± SEM.

**Figure 3:**
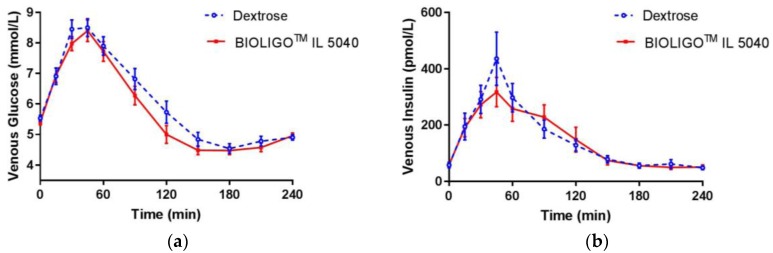
Post-prandial glycemic response in healthy adults (**a**) venous glucose (**b**) venous insulin concentration (Study 1). Data are mean ± SEM.

The changes in breath hydrogen concentration in response to dextrose and BIOLIGO^TM^ IL5040 IMO, over 24 h are shown in Figure 2. There was no significant difference between IMO and the control at all time points. Post-prandial glycemic (Figure 3a) and insulinemic response (Figure 3b) to dextrose and BIOLIGO^TM^ IL5040 IMO, showed no significant differences over 4 h. 

Glucose iAUC in the first two and four hours was ~11% and ~15% lower, respectively, for IMO compared to the control (Table 4). These differences were not large enough to reach statistical significance, but the difference in glucose iAUC over 4 h, neared significance (*p* = 0.058). Glucose iAUC from 2–4 h was significantly lower for IMO compared to the control (*p* = 0.008). There were no significant differences between treatments, for any of the other glucose and insulin iAUC. In study 2, the glucose iAUC 0–2 h means (± SEM) were not significantly different from one another (BIOLIGO™ IL7010: 201.5 ± 25.5; BIOLIGO™ IL5040: 181.3 ± 23.4; Dextrose: 191.3 ± 22.2: *p* = 0.662).

**Figure 4:**
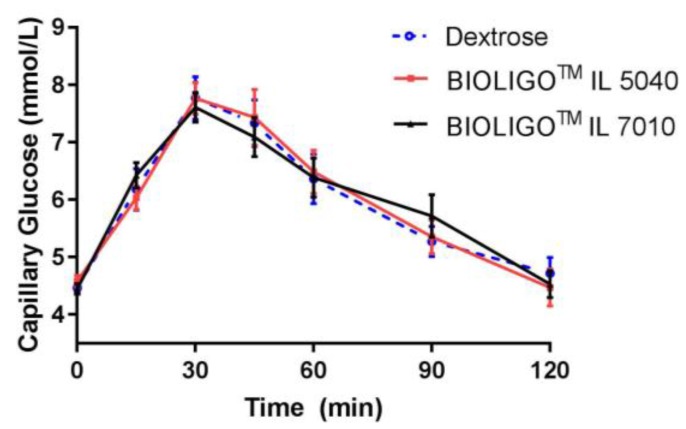
Post-prandial glycemic response to BIOLIGO^TM^ IL5040 and BIOLIGO^TM^ IL7010 IMO (Study 2). Data are mean ± SEM.

Following consumption of two IMOs, there was no significant difference in capillary blood glucose concentrations compared to dextrose, in healthy adults (Figure 4). The compositional differences between two IMOs (Table 5 did not impact their effects on glycemic response.

## 4. Discussion

Sweeteners provide taste and rheological attributes (texture, flavor, preservative, and color) in food and beverages [17]. Although lowering post-prandial blood glucose response is considered as a physiological benefit, sugar-reduced products are less accepted by consumers. Thus, food manufacturers utilize various sweeteners based on their origin (natural or synthetic), technological function (taste and fillers), texture (powders and syrups), and nutritional value (caloric and non-caloric). Low digestible carbohydrates such as polyols or sugar alcohols are the most commonly used sugar substitutes, due to their low-caloric value, low glycemic response, and non-cariogenicity. Despite their health benefits, sugar alcohols may have transient gastrointestinal effects at excessive intakes.

Emerging nutritive sweeteners include rare sugars and oligosaccharides, and are appealing due to their natural source [17]. Oligosaccharides such as fructooligosaccharides (FOSs), and isomaltooligosaccharides (IMOs) are used as either partial or full-sugar replacers in food formulations [18]. The majority of the studies that tested IMOs in Asian populations had conflicting evidence on their digestibility and fermentability. Glycemic response is an indicator of carbohydrate digestibility. Fully digestible carbohydrates such as dextrose produce a rapid rise and fall in blood glucose. Insulin is released in response to initial blood glucose rise and causes it to fall. Non-digestible carbohydrates containing glucose show negligible glycemic response, while partially absorbed polyols do not cause increased blood glucose levels. Previous studies, with different commercial IMOs, are inconsistent and reported mixed results (bifidogenic and no impact on blood glucose breath hydrogen) suggesting IMO to be partly digestible and partly fermentable [19].

This is the first study to evaluate gastrointestinal tolerance and glycemic response of IMO in a healthy population. In this short-term study, BIOLIGO™ IL5040 IMO at 68.5 g/day on an as-is basis (25 g/day pure IMO on a dry basis where IMO content is defined as in Section 2) was well-tolerated as demonstrated by having no impact on the composite gastrointestinal symptom score, and frequency of individual intestinal symptoms. Bouhnik et al. [20] tested IMO, among other oligosaccharides, at 10 g/day for one week in healthy adults and reported no significant changes in four intestinal symptoms (excess flatus, bloating, borborygmi, and abdominal pain). We measured breath hydrogen to assess fermentability of IMO over 24 h. Although breath hydrogen increased numerically between 6–12 h, there was no significant difference between IMO and dextrose over 24 h. All participants were provided with a standardized (low fiber/low dairy) lunch, snack and dinner before and during the test days, to avoid the dietary impact on breath hydrogen. Earlier studies [21] examined breath hydrogen response to IMO up to 25 g/day for 7 h in healthy adults and reported no effect on breath hydrogen, reflecting insufficient evidence of fermentation. 

In another study [15], increase in blood glucose indicated IMO to be highly glycemic. In both clinical studies presented in this paper, BIOLIGO^TM^ IL5040 and IL7010 IMO dose-matched for 50 g total carbohydrates showed similar glycemic response compared to dextrose. Though iAUC for venous blood glucose was significantly lower than dextrose beyond 2 h, venous insulin response to BIOLIGO^TM^ IL5040 showed no significant change compared to the control. The tested IMOs differed compositionally in Study 2, with BIOLIGO^TM^ IL7010 having a higher average DP than BIOLIGO^TM^ IL5040. However, this difference in the degree of polymerization between the two IMOs did not affect their digestibility and was shown to be both fully digestible and hence caloric.

The majority of the IMO studies assessed Bifidobacteria in Asian populations [22,23], while no Bifidogenic effect was observed in European men and women [20]. The studies that showed changes in Bifidobacteria, were not well-designed, with no proper control groups. Positive effects of IMO on stool frequency and stool weight were reported in constipated populations [24,25,26]. Although the present Study 1 did not assess changes in fecal microbiota, the low fermentation of the IMO in study 1 suggests that it would not provide a bifidogenic effect. Further research is necessary to confirm changes in fecal microbiota and determine if this product affects bowel habits.

According to the UK Food Standard Agency, commercial IMO is a novel ingredient and glycemic or digestible carbohydrate [27]. The evidence suggests that IMO is highly digestible with a small residual portion reaching the colon (estimated at 10%) and affecting the microbiota. Certain populations, such as Asians may experience beneficial changes in the microbiota and changes in laxation for constipated individuals, with a sufficient dose, but no evidence exists to confirm these effects in non-Asian populations. Variability in IMO compositions from different manufacturers may be one of the reasons for the conflicting evidence on digestibility and fermentability. 

Although we have demonstrated that the tested IMOs are digestible with high gastrointestinal tolerance, these two clinical studies have a few limitations. The current studies were conducted in a Caucasian population. Additional research in an Asian population may provide insights into population-specific differences in digestibility and fermentability. Secondly, these were acute studies, and microbial changes in stool samples were not measured. Long-term evaluation would be needed to determine whether these ingredients have a prebiotic effect or impact laxation patterns. However, due to the high digestibility and low fermentability reported in the present studies, a prebiotic effect is unlikely.

## 5. Conclusions

BIOLIGO^TM^ IMOs are well-tolerated as demonstrated by the lack of adverse gastrointestinal symptoms and they have no effect on breath hydrogen (an indicator of fermentability). Additionally, these IMOs are caloric sweeteners based on the glycemic and insulinemic response in healthy adults. Further studies are needed to determine the postprandial effects of larger doses of IMO on blood glucose, gastrointestinal tolerance and gut microbiota over longer durations.

## Figures and Tables

**Table 1:** Subject demographics in study 1.

Mean ± SEM	Participants (*n* = 26)
Age (years)	39.9 ± 1.9
Gender (male/female)	16/15
Weight (kg)	76.5 ± 2.4
Body mass index (kg/m^2^)	25.8 ± 0.4

**Table 2:** Subject demographics in study 2.

Mean ± SEM	Participants (*n* = 10)
Age (years)	33.9 ± 3.5
Gender (male/female)	4/6
Weight (kg)	73.1 ± 4.8
Body mass index (kg/m^2^)	26.3 ± 0.9

**Table 3:** Frequency of scores ≥2 ^a^ on individual gastrointestinal (GI) symptoms (Study 1).

GI Symptoms	Dextrose *n* (%)	BIOLIGO^TM^ IL5040 IMO *n* (%)	*p*-Value ^b^
Nausea	1 (3.8)	0 (0.0)	0.68
Bloating	3 (11.5)	2 (7.7)	0.43
Rumblings	4 (15.4)	4(15.4)	0.89
Flatulence	4 (15.4)	5 (19.2)	0.56
Abdominal pain	1 (3.8)	1 (3.8)	1.00
Diarrhea	2 (7.7)	2 (7.7)	0.23

^a^ Scoring system was 0 = none, 1 = no more than usual, 2 = somewhat more than usual, and 3 = much more than usual. ^b^ *p*-values derived from repeated measures analysis using the GEE method with subjects included as a random effect. IMO: isomaltooligosaccharides.

**Table 4:** Incremental area-under-curve (iAUC) for glucose and insulin (Study 1).

Parameters	Dextrose	BIOLIGO^TM^ IL5040 IMO	*p*-Value
Glucose iAUC (0–2 h)	211.4 ± 19.5	189.0 ± 18.1	0.189
Glucose iAUC (0–4 h)	230.0 ± 22.8	194.8 ± 20.1	0.058
Glucose iAUC (2–4 h)	18.6 ± 7.2	5.9 ± 3.1	0.008
Insulin iAUC (0–2 h)	21855 ± 3573	20068 ± 2956	0.743
Insulin iAUC (0–4 h)	25378 ± 3674	22781 ± 3494	0.354
Insulin iAUC (2–4 h)	3523 ± 1119	2712 ± 1004	0.290

iAUC: incremental area under the curve; IMO: isomaltooligosaccharides; Glucose iAUC ((min × mmol/L); insulin iAUC (min × pmol/L)); Data are mean ± SEM; *p*-values derived from repeated measures analysis of variance (ANOVA).

**Table 5:** Compositional differences of tested IMOs.

IMO	DP 1–2 (% Dry Basis)	DP 3–8 (% Dry Basis)	DP 9+ (% Dry Basis)	IMO Content * (% Dry Basis)
BIOLIGO^TM^ IL5040	43.0	53.3	3.3	50
BIOLIGO^TM^ IL7010	9.2	77.4	13.5	70

DP: Degree of polymerization. BIOLIGO^TM^ IL5040 and BIOLIGO^TM^ IL7010 are syrups with an average of 24% water content, as-is. * IMO content, in this paper, is defined as the sum of IMO components from DP2 to DP7 (isomaltose, panose, isomaltotriose, isomaltotetraose, isomaltopentaose, isomaltohexaose, isomaltoheptaose).
